# Operational challenges and considerations for COVID-19 research in humanitarian settings: A qualitative study of a project in Eastern Democratic Republic of the Congo and South Sudan

**DOI:** 10.1371/journal.pone.0267822

**Published:** 2022-06-30

**Authors:** Jennifer Majer, Kelechi Udoh, Arsene Beleke, Dugisye Ahmed, Deepak Kumar, Aimee Summers, Mija Ververs, the IMC-CDC COVID-19 Research Team, Iris Bollemeijer, Shannon Doocy

**Affiliations:** 1 International Programs Department, International Medical Corps, Los Angeles, California, United States of America; 2 Programs Department, International Medical Corps, Juba, South Sudan; 3 Programs Department, International Medical Corps, Goma, Democratic Republic of Congo; 4 Emergency Response and Recovery Branch, Centers for Disease Control and Prevention (CDC), Atlanta, Georgia, United States of America; 5 Department of International Health, Johns Hopkins Bloomberg School of Public Health, Baltimore, Maryland, United States of America; CDC, UNITED STATES

## Abstract

Since the beginning of the COVID-19 pandemic, much research has been conducted globally, but relatively few studies have been carried out in complex emergency settings that pose numerous operational challenges. We conducted a qualitative study to explore the barriers and enablers of a COVID-19 cohort study conducted in South Sudan and Eastern Democratic Republic of the Congo, to inform future research on COVID-19 and infectious diseases in humanitarian settings. We used a case study design embedded within the original prospective cohort study. Qualitative data was collected through four health facility assessments, 28 key informant interviews, and a focus group discussion. Data were analyzed using a manual thematic analysis approach and summarized against four primary themes: testing challenges and enablers, perceptions and attitudes towards COVID-19, national health system considerations, and study management considerations. Findings suggest most of the challenges affecting the cohort study were not specific to COVID-19 research but have been a feature of previous infectious disease research carried out in complex emergencies. However, the pandemic has exacerbated certain problems. The high proportion of travellers enrolled due to testing mandates, stigmatization of infected individuals linked to the heavy global focus on COVID-19, strained resources during waves of increasing infections, and remote management requirements all negatively impacted the cohort study. Factors that facilitated the research included proactive management, data quality oversight procedures, and strong collaboration with national health stakeholders. The global impact of COVID-19, its high public profile, and specific pandemic policies pose further operational challenges for research in already complex humanitarian settings. Future studies could plan mitigation measures that include flexibility in staffing and budgets, strategies to expand testing, and early partnerships with local organizations and health authorities.

## Introduction

The COVID-19 pandemic has posed unprecedented challenges to public health researchers, given widespread changes to workplace policies and procedures of scientific institutions [1–3]. Among the many complexities, researchers have had to adapt to new protocols for remote management in the design and implementation of studies. These changes are particularly challenging for research carried out in low-resource settings that lack reliable technology, adequate human and financial resources, and health system capacity.

Research on COVID-19 in Sub-Saharan Africa and in complex emergency settings is particulalry lacking. Most African research on COVID-19 has been clustered in South Africa, Egypt, and Nigeria, translating to a paucity of evidence from humanitarian settings where populations often present with different risk profiles and health characteristics and where health services are more limited [4]. This may be partly attributable to specific challenges of pandemic research, as well as difficulties that have been previously identified in designing and managing robust studies in emergency settings [5–8]. However, little documentation exists describing the realities of COVID-19 research in these types of environments.

This case study describes the operational experience implementing an observational study on COVID-19 in two humanitarian settings in Sub-Saharan Africa. It is based on a prospective cohort study conducted by the Johns Hopkins Bloomberg School of Public Health (JHSPH), International Medical Corps (IMC), and the U.S. Centers for Disease Control and Prevention (CDC) in North and South Kivu provinces in Eastern Democratic Republic of Congo (DRC) and Juba, South Sudan (SSD). In conducting this case study, the specific objectives were 1) to inform and contextualize the findings of our cohort study and 2) reflect on ways in which the COVID-19 situation impacted the study in these humanitarian settings. Several themes emerged as key operational constraints and enabling factors for our study, many of which were not specific to COVID-19 research but exacerbated by the pandemic. By documenting this experience, we aim to promote and inform future research during complex emergencies both during and after the pandemic.

## Methods

This study employed a case study design, embedded within a prospective cohort study described above. Briefly, five health facilities served as enrollment sites for the cohort study, including a COVID-19 treatment center in Juba, SSD and four health facilities in DRC. Patients receiving inpatient or outpatient case management of COVID-19 as part of the routine COVID-19 response in each country between December 2020 and June 2021 were eligible for enrollment in the study. Consenting patients were registered and followed to discharge. Data collected from the patients included demographics, current and past medical history, and repeated clinical measurements to evaluate risk factors associated with hospitalization and death. The study protocol and results are fully described in companion papers [9, 10].

This case study primarily utilized qualitative data collected through 1) Key Informant Interviews (KIIs) with clinicians, laboratory staff, epidemiologists, and focal points from various response agencies and COVID-19 taskforces; and 2) a Focus Group Discussion (FGD) conducted with IMC staff involved in the study. We also reviewed quantitative data from the cohort study, health facility assessments (HFA), and secondary sources including published databases on testing and cases, which was used as supporting evidence and triangulated with the qualitative data where relevant. Reflexivity was an important component in designing and conducting the case study, and in analysis and interpretation of the data. The researchers come from diverse professional backgrounds (as nutritionists, epidemiologists, medical doctors, and researchers) and countries of origin (DRC, Ethiopia, Netherlands, Nigeria, Pakistan, and the United States). As a team we brought a range of perspectives, contextual experiences, and awareness of cultural nuances, strengthening the quality and validity of the study.

Qualitative data for the case study was collected at two periods, at the beginning of enrollment for the cohort study in December 2020 and the end of enrollment in June 2021 (Table 1). A total of 11 key informants (KIs) participated in the first round and 17 KIs in the second round. In the first period, KIIs were conducted with COVID-19 frontline healthcare providers to explore issues around patient characteristics and outcomes, components and quality of patient care, availability of equipment and supplies, and provider capacity. The HFA was simultaneously conducted in each of the participating health facilities (4 in DRC, 1 in SSD) using a structured observation checklist that documented available human resources, equipment, and supplies considered to impact COVID-19 case management. A second round of KIIs was conducted in June 2021 at the end of data collection for the cohort study. KIs included stakeholders involved in surveillance and testing, patient care, and laboratories to understand COVID-19 epidemiological trends and surveillance, case presentation, care seeking behavior, potential risk factors, changes in national COVID-19 testing and case management protocols and aspects of quality of care, as well as the COVID-19 testing situation. A FGD was also conducted with the research teams in both countries to explore internal and external challenges and enablers.

**Table 1:** Summary of data collection and respondent groups.

Method	Stakeholder category	Number of interviews	Positions
**Key informant interviews (KII)**	**COVID-19 service providers**	Pre-study: 7 DRC, 4 SSD	Medical doctors
		Post-study: 2 DRC, 5 SSD	Nurses
	**COVID-19 surveillance experts**	Post-study: 3 DRC, 2 SSD	Epidemiologists
			Medical doctors
	**COVID-19 laboratory experts**	Post-study: 2 DRC, 3 SSD	Laboratory managers
			Virologists
			Laboratory technicians
**Focus group discussion**	**IMC research implementation team**	Post-study: 2 DRC, 3 SSD	Research managers
			Program Director
			Medical Coordinator
			M&E Coordinator

Purposive sampling was used to identify KIs on the basis of their experience with the COVID-19 response in each study location. KIs held positions with agencies including IMC, CDC, Ministries of Health of DRC and South Sudan, and public and private laboratories processing COVID-19 tests. All KIs possessed more than 10 years of experience in their respective fields and were deemed to have a high level of knowledge about the topics included in KII guides. All IMC staff in DRC (2) and SSD (4) involved in supervising research implementation were invited to participate in the FGD. IMC headquarters (HQ) study team members conducted FGDs virtually with the research implementation teams in DRC and SSD. Unique KII and FGD guides were developed for each cadre of respondents (Table 2).

**Table 2:** Summary of key informants and areas of focus during interviews.

Key Informants	Focus Areas
**COVID-19 service providers**	Exploration of temporal changes and differences in clinical factors across the study settings that may have influenced the study results, including: • Case management practices • Case presentation • Care seeking • Protocols • Quality of care
**COVID-19 surveillance experts**	Exploration of temporal changes and differences across study settings related to data quality, to be considered when interpreting the study results, including: • Surveillance strategies and effectiveness, especially the representativeness, completeness, and timeliness of case identification • Testing strategies and effectiveness • Barriers and enablers of surveillance and testing
**COVID-19 laboratory experts**	Exploration of temporal changes and differences across study settings related to processing of COVID-19 tests, including: • Laboratory testing capacity • Volume of tests conducted • Supply and demand for tests • Processing in private and public laboratories
**IMC research implementation team**	Consultation with IMC research staff in DRC and South Sudan, focusing on their similar and diverging perspectives on operational and contextual challenges in carrying out the study, including: • Relationship with and involvement of study stakeholders • Enrollment and follow-up challenges • Data quality management • Staffing and resource allocation • Study logistics • Procurement and supplies, communication

The data collection tools were developed in English by JHSPH, IMC, and CDC study staff; DRC data collection tools were translated into French. The tools were pre-tested by expert reviewers and piloted prior to each phase of data collection. The IMC research managers conducted HFAs and KIIs in person, with research assistants as notetakers and voice recorders used with consent. Informed consent was obtained verbally prior to data collection in the presence of the research assistants, and documented through confirmation of consent on the paper forms. A daily debrief was conducted among the research team to discuss emerging findings, review notes, and identify areas of further inquiry. All KIIs and FGD transcripts and notes written in French were translated to English and translation verified. Transcripts developed from recordings and interview notes were typed and reviewed for both quality and consistency.

KIIs and FGD data were analyzed using a thematic analysis approach [11, 12]. First, four authors jointly identified a sample of transcripts and notes representing different stakeholders; then transcripts and notes were independently and manually analyzed by inductively identifying codes (open coding) and then combining the codes into sub-themes, themes, and topics (axial coding) [13, 14]. Following the initial analysis, all authors reviewed disparities in initial codes, sub-themes, themes, and topics, to reach consensus on definitions. The resulting definitions were input into a coding guide which was used in the second phase to deductively code transcripts and notes.

Throughout the analysis process the codes, sub-themes, and themes were adapted and the coding guide was updated with emergent codes, sub-themes, and themes. All coded transcripts were reviewed for quality and consistency with the coding guide before moving to the second phase where major themes were prioritized. It was agreed a priori to restrict potential themes to the two topic areas related to study implementation: COVID-19 surveillance and testing, and research operational challenges and enablers. Eligibility was further restricted to themes referenced at least once in each country, leaving twenty-one eligible thematic areas (Table 3).

**Table 3:** Restricted list of topics and thematic areas considered for major themes.

Topic	Thematic areas
**COVID-19 Surveillance and Testing**	• Testing quality • Data quality • COVID-19 surveillance approaches • COVID-19 surveillance system enablers and barriers • Result outcome • Travelers • Community members • Handling of testing for influential people (VIPs) • Geographic residence • Professional status
**Research Operational Challenges and Enablers**	• Low coordination among study stakeholders • Low human resource management and support • Humanitarian context • Health system challenges • Enrollment challenges • Data quality and missing data • High coordination among study stakeholders • Study staffing and capacity building • Operations and logistics • Data quality enablers • Enablers of enrollment

In the third phase, the thematic areas were merged based on conceptually related ideas and the authors reached consensus on the top four primary influences (challenges or enablers) of the study which included: 1) testing challenges and enablers; 2) perceptions and attitudes toward COVID-19; 3) national health system considerations; and 4) study management considerations. In the fourth phase, major findings from the interviews were summarized by theme and sub-theme as applicable. The codes relevant to each thematic area were systematically reviewed for each set of notes and transcripts, and the perspectives of respondents across each country and stakeholder groups were compared.

The qualitative study and the original cohort study, including data collection tools and consent protocols, were reviewed and approved by the Johns Hopkins University Institutional Review Board, the South Sudan Ministry of Health (MoH) Ethics Committee, University of Kinshasa School of Public Health, and the US CDC. The study is registered with ClinicalTrials.gov (NCT04568499).

## Results

### Testing challenges and enablers

#### Sub-optimal laboratory capacity for testing identified as a barrier

Low numbers of persons tested was raised by KIs as a chronic challenge during the study period. Clinicians, laboratory staff, and research team members highlighted in interviews how shortages in testing supplies and insufficient processing capacity reduced the public’s access to testing, which in turn affected the volume of enrollments. In DRC, we encountered delays in receiving patients’ test results due to sub-optimal in-country laboratory processing capacity, which meant suspected cases often became ineligible for enrollment. This affected 213 prospective enrollments, or 20.8% of the sample of eligible participants in DRC, mainly in the first two months of the study period. In both countries, KIs reported that insufficient quantity and over-centralization of equipment and supplies (e.g., test kits) slowed the processing of samples.

*“The supply was not adequate. Stockouts of tests, collection kits, transport media, etc. were very frequent, especially before February 2021. To remedy this situation, the system had opted for targeted screening (cases meeting the definition of suspected cases, and other patients with chronic diseases).” (KI, MOH, DRC)*

*“We seriously need to build up the capacity of testing, to expand it, and maybe we have some automated kind of testing so that we increase the samples for extraction and remember our people are using the manual extraction so if we can have the automated one so that a good number of samples are extracted… I think it will assist us a lot.” (KI, MOH, SSD)*

**Fig 1:**
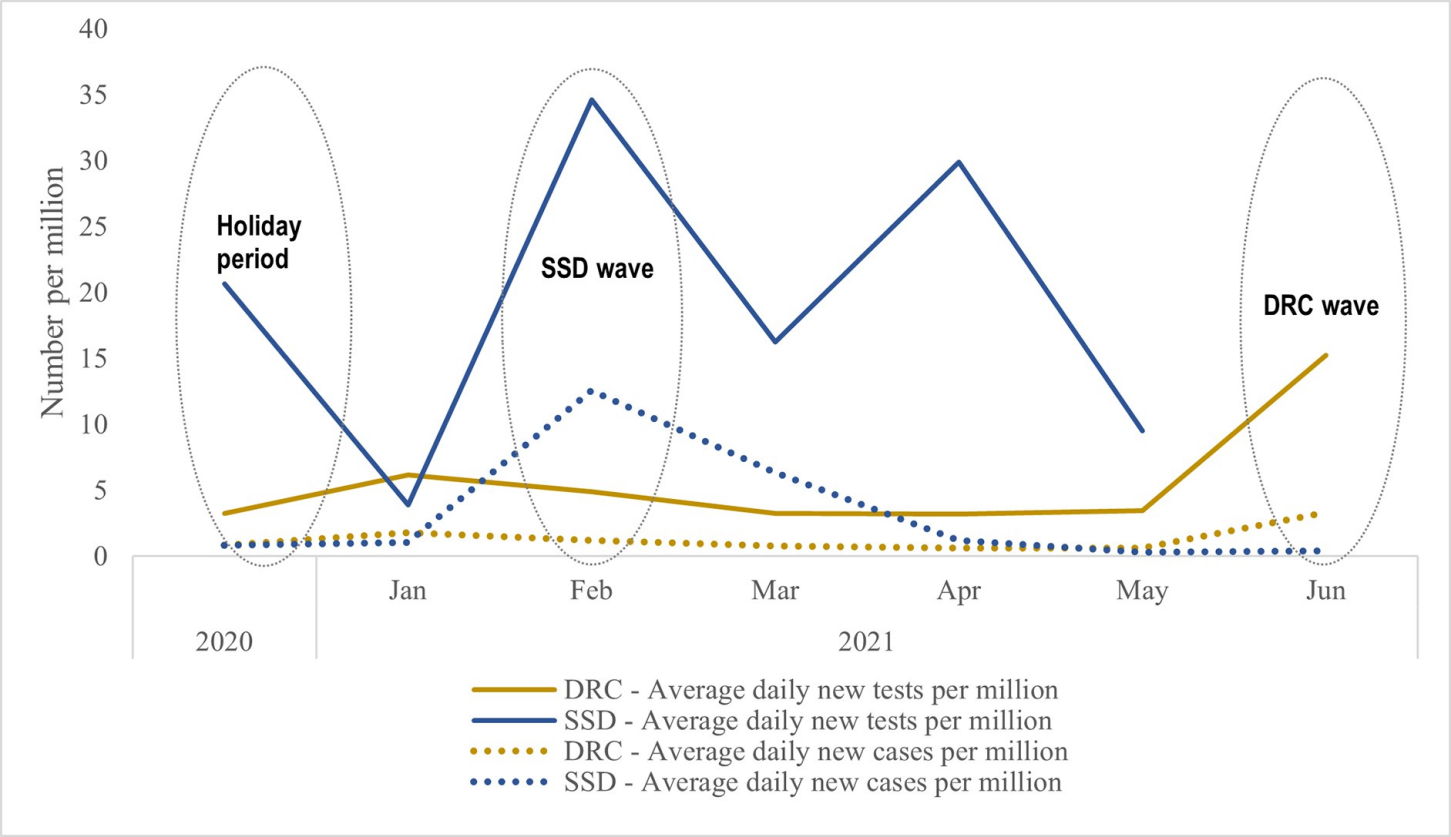
COVID-19 testing and case trends in the Democratic Republic of the Congo and South Sudan, December 2020-June 2021. SSD = South Sudan. The Democratic Republic of the Congo = DRC. Waves = Time periods with increases in infection. Holiday period = Time period from December to January. Source: Reproduced from data originally published by Hasell et al. in Nature [15].

Review of national testing data confirms COVID-19 testing rates were extremely low in each country during the study period, with an average of only 6 and 18 tests per million persons per day recorded in DRC and SSD, respectively, during the study period [15] (Fig 1). Trends in new cases identified–and thus eligible enrollements–mostly corresponded to peaks and troughs in numbers of persons tested. In SSD, testing volume and new cases increased during the country’s second wave of increased infections, reaching an average of 848 tests per day (77 per million people) in February 2021, while DRC reported an increase in testing and new cases in May and June 2021 which corresponded to the beginning of the third wave in DRC.

#### Expansion of testing capacity during the study period identified as an enabler

Both governments adjusted their testing protocols in ways that expanded access to testing around the time the study began in December 2020. Changes in national policies that allowed for reverse transcriptase polymerase chain reaction (RT-PCR) testing in non-government facilities became an important enabler of COVID-19 testing in SSD, which helped increase study enrollment. While testing was initially restricted to government facilities in both countries, by January 2021, several private laboratories were commissioned in SSD to provide testing for international travelers, which allowed government laboratories to focus testing resources on persons who were symptomatic or contacts of confirmed cases, thereby improving testing availability.

*“So, they [MOH] expanded testing for alerts and suspected cases at the public laboratory. Once that component [traveler screening] was removed from the public laboratory, they were able to increase the number of tests… In South Sudan, the introduction of private laboratories turned out to be the big factor. It actually played the biggest role (in improving testing access) because (the private laboratories)… really helped ramp up testing a lot.” (KI, Implementing Partner for COVID-19 response, SSD)*

The referral laboratories run by the government were initially located in the capital cities of DRC and SSD, and samples had to be transported to Kinshasa and Juba, respectively. The number and geographic distribution of laboratories were later expanded to other provinces/states, a factor that increased access to testing, particularly among potential cases in the DRC cohort. The governments also leveraged existing testing systems (PCR assays) such as those for Ebola Virus Disease and HIV to increase testing capacity. Also, in DRC, introduction of rapid diagnostic tests (RDTs) reduced not only the time required to confirm COVID-19, but reduced the cost per test and allowed for more efficient use of supplies required for RT-PCR testing as well. RDTs were distributed to some health facilities in January 2021 and were available for free or at subsidized cost.

*“Before January 2021, the number of tests performed was very low because only PCR was validated for diagnosis. From mid-January 2021 to date, the number of tests carried out has increased significantly by increasing the capacity of the mobile laboratories and by introducing rapid tests as a means of diagnosis.” (KI, MOH, DRC)*

#### Differential testing among at-risk target populations identified as a barrier

To mitigate frequent stock outs and manage limited resources, both SSD and DRC used targeted testing and surveillance strategies focusing on those more likely to be positive (e.g., symptomatic people and close contacts of cases). A negative COVID-19 test was required on arrival in DRC, SSD, and many neighboring countries for international travel by air and across land borders. In DRC, the testing requirement also applied to in-country travel and in SSD, KIs noted that a negative COVID-19 test was required to exit post-travel quarantine. Those interviewed generally considered travel related testing strategies to be a facilitator of testing, but also a source of disparity in coverage of testing where travelers accounted for most of the tests performed, especially around the Christmas/New Year holiday period.

*“You remember the Christmas season? …when the Christmas season came, yes, everybody wanted to travel. That’s why there were many travelers in December (2020).” (KI, Private Laboratory, SSD)*

Additionally, participants described the link between higher socio-economic status (SES), travel, and access to testing. In DRC and SSD, KIs remarked that supplies and services for paid tests (i.e., private laboratories) were more readily available, which was not the case for free tests at government laboratories that experienced stockouts. For example, in SSD, the private labs testing travellers retained five-to-six months’ worth of stock to guarantee availability, which resulted in better access to testing among those who could pay. This also increased the potential for travelers and those with higher SES to be enrolled in the study, given they had better access to testing and were more likely to receive test results.

*“Though testing remained free for patients in hospitals or persons visiting health facilities, samples from travelers who pay were often prioritized by testing structures. This contributed to challenges getting tests for patients including those enrolled in the study.” (FGD, IMC South Sudan field study team)*

Participants also related higher SES with demand for and the higher rate of testing among certain professional categories of workers [e.g., Non-Governmental Organizations (NGO) and health workers] that often underwent mandatory testing following exposure at work and/or voluntarily testing because of high COVID-19 awareness.

*“For us here, everyone was tested because we are part of the response, so I guess we could say people in the response are more willing to be tested because they know what is at stake.” (KI, Implementing Partner, SSD)*

A variety of demographic factors were reported as influencing the profile of individuals tested in each country. For children, KIs described the unwillingness of families to travel with children during the pandemic and the need for a caregiver to bring the child to a facility as reasons for relatively low testing rates. KIs from SSD reported in both the HFA and end-line interviews that older adults and females were comparatively under-represented in testing. Explanations for the lower proportion of females seeking COVID-19 tests compared to males were offered in terms of differential exposure in work, social life, and travel. KIs also noted that men were more likely to work outside the home and travel.

*“No, I think it [the lower proportion of females testing] should be due to some other… cultural kind of thinking. They normally say: ‘The woman has to stay in the home as the man rushes for hustle’ and the level of caring for a lady is very poor, until when it reaches a critical situation.’” (KI, MOH, SSD)*.

### National health system capacity

The health systems in both countries faced challenges mobilizing sufficient supplies for quality care. KIs described in both the HFA and end-line interviews the limited capacity of health facilities to care for and follow up on COVID-19 patients. Problems included lack of consistent food and nutrition support for isolated outpatients and quarantined close contacts; shortages in basic equipment such as respirators; insufficient human resources for advanced care such as ventilation; and lack of tests for co-morbid conditions. These gaps at health facilities were similarly identified in our study sites during the HFA (see clinical progression companion paper for further description of the capacity of the health facilities [10]).

KIs remarked that restocks of testing and treatment supplies were insufficient and did not match the needs of increasing case numbers, especially during waves of increased infections. Moreover, due to lack of COVID-19 testing supplies, the governments’ free testing and healthcare policies were not implemented. The limited access to services among patients who could not afford the fees, may have deterred care-seeking and subsequently opportunities for study enrollment.

*“Stock outs compromised care quality and discouraged patients and their families from seeking care at health facilities. Having to pay yourself for medicines in public pharmacies did not make things easier. In desperation people will go to prayer houses and traditional healers for solutions especially ones they perceived faster.” (FGD, IMC DRC field study team)*

Gaps in government human resources were observed in both countries in ways that impacted the study. KIs attributed these challenges to an inadequate number of staff as well as sub-optimal staff compensation and consequently low motivation. The financial incentives put in place to compensate staff for COVID-19 risk in both settings were low and frequently unpaid which culminated in a health worker strike in May 2021 in SSD. Those interviewed in SSD highlighted the absence of a MoH policy to care for staff if they became infected while engaged in the response. Inadequate number of trained laboratorians and other health staff was further identified as a key barrier to COVID-19 testing, specifically, in government laboratories in both countries.

*“The issue of human resource is another challenge as the country has a lot of challenges in terms of retaining the staff. Once you train them, there is high rate of attrition, and the lab is left sometimes empty. Nobody to come and work as they go for the hot cakes such as the private clinics which normally pay better.” (KI, MOH, SSD)*

The accuracy and timeliness of routine COVID-19 data produced by the health system also posed challenges for the study, as enrollment procedures relied on surveillance and laboratory data for the identification of COVID-19 cases. Logistical problems were experienced in DRC, where slow transporting and processing of samples, inadequate testing materials and lack of communication were reported as the main factors hindering functionality of the disease surveillance system. KIs in SSD cited sub-optimal MoH reporting guidance and lack of standardized tools that persisted through the study period, leading to incomplete or delayed information on positive cases.

*“The first reason; there are national tools that are supposed to be used for COVID-19 across the country [and] unfortunately these tools have not been disseminated to sub-national levels adequately. Once partners send us these aggregated numbers like the tests run and number of positive cases, they think their role is done. That is why we have incomplete data but also for some locations COVID-19 is just like any other additional thing they’re doing already.” (KI, Implementing Partner, SSD)*

Systems-level challenges were coupled with intentionally incomplete or inaccurate information shared by patients and health facilities and laboratories, which reduced the pool of reachable patients for study enrollment.

*“We have issues with data especially initially because the labs were giving people the form to fill and you know they [the patients] fill the part they’re comfortable with, as well as wrong information like about their [phone] number.” (KI, MOH, SSD)*

### Perceptions and attitudes towards COVID-19

Attitudes towards the COVID-19 situation were characterized by high levels of stigmatization and mistrust that persisted throughout the cohort study period. Disbelief in the existence or severity of COVID-19 was prominent during interviews conducted for the HFA, prior to large increases in cases and deaths in the first half of 2021.

*“They [patients with COVID-19] are also having doubt because they said they don’t even have any signs, but later they… are positive, that’s the problem. [They consider] this pandemic here in Africa is not something which is even… serious. You know in Europe, it’s the one even killing people, but for us here our immune system is very strong. You find even when you catch [the virus], you [do not] even show any symptoms.” (KI, Case Management Officer, SSD)*

Notably, KIs interviewed in May and June 2021, at the end of the study, no longer referenced disbelief in the virus as a widely held attitude among the public. Instead, fear and shame became more common reactions to the virus. Those interviewed emphasized the stigmatization that followed the rise of cases in both countries, and the negative impact on symptomatic individuals seeking testing and health care as well as study enrollment. Those interviewed also believed the reluctance to test despite symptoms was exacerbated by the isolation measures undertaken among positive patients, as well as policies that prohibited families from visiting sick patients in the infectious disease unit (IDU) in SSD.

*“It comes with um, you know, a bit of stigma once you have it. From the early days, you know, people fear, people don’t want to come up openly. If you have cough, you may not want to say I want to go and test because I have cough because you fear people around you may say no, no we don’t want to stay near this person. So, uptake you know for testing is a huge problem.” (KI, MOH, SSD)*

*“[A patient believes] if he goes [to the IDU], he will die. And of course, everyone will refuse to enter the red zone. If you have entered IDU either you improved or died. And when you are in such place, also it is not open to public even relatives will not visit you as you want and so on.” (KI, Clinician, SSD)*

Fear and stigmatization led to highly negative reactions to positive test results among some individuals. The research team often encountered refusals to enroll in the cohort study among individuals who were angry and rejected their results.

*“Stigma was a big issue accounting for refusals. People were really angry about their results and would scream at the case management team. People would drop the call from the research nurses or get aggressive. They wouldn’t accept they had COVID-19 or participate in any of the research.” (FGD, IMC South Sudan field study team)*

The reluctance of COVID-19 cases to be followed up is reflected in the challenges faced during study enrollment. KIs remarked that most members of the general public were reluctant to get tested and seek care, even if they were symptomatic. This could have introduced bias if demand was differential in various population sub-groups, because it could skew the distribution of individuals being tested (e.g., by sex, age group). Reluctance to test was fostered primarily by low perceived risk.

*“People seeking test (for) symptoms is not a big number. I witnessed one or two people who came, and they wanted to screen because they said they don’t feel okay, they have some symptoms like COVID-19.” (KI, MOH, SSD)*

KIs in SSD acknowledged the governments’ efforts to improve risk communication and community engagement (RCCE) during the country’s second COVID-19 wave to increase testing and careseeking behaviors. However, low awareness and interest in COVID-19 testing remained a barrier. Those interviewed believed efforts were still overly focused within major cities and certain regions. Insufficient RCCE limited awareness of the free testing options that were available.

*“Yes, there are these different components to the response but for the risk communication component I don’t think they have done a very good job. I am sorry to say that [especially] in terms of demand for testing in the community. So, people with comorbidities even if they have diabetes and really wanted to be tested, I do not think that information got to them, so I wouldn’t say that people with [co-]morbidities are more likely to be tested.” (KI, MOH, SSD)*

### Study management considerations

Research project management practices were considered highly influential in the quality of the cohort study, in terms of enrollments and data quality. KIs and study staff considered key constraints to be lack of human resource flexibility and operational limitations in collecting certain clinical data, while they highlighted as good practices the data quality assurance procedures and strong collaboration with local stakeholders.

#### Human resources

Staff who oversaw implementation pointed to several staffing factors that affected the study. While the study in SSD relied primarily on IMC staff (two dedicated research nurses) for data collection, in DRC fewer research nurses were hired compared to the larger geographic areas covered. As a result, in DRC a larger portion of the data was collected by MoH staff who were incentivized to record patient information for the study. The research team in DRC faced greater challenges with data quality and reporting timelines due to overstretched MoH staff.

Staffing levels became a particular challenge during COVID-19 waves. Throughout the cohort study, low levels of testing meant the number of eligible participants to enroll (on average less than 2 cases per day in each setting) could be easily handled with the study’s original staffing structure. However, existing research staff quickly became overwhelmed when new waves of infections occurred in SSD (February 2021) and DRC (May 2021) and they were unable to contact all positive cases within several days of receiving a positive test result as the protocol specified. The SSD research team initially sought to hire temporary staff to increase enrollment rates, but internal procedures prolonged the recruitment process. By the time job advertisements were posted and interviews were conducted, the wave had ended. Staff from other departments were seconded to support enrollment and follow-up on a part-time basis, however, there were many other competing demands for health staff and the approach was not optimal. Study staff believed the fixed number of human resources, especially during the peak period of COVID-19 cases, resulted in incomplete enrollment of eligible cases.

*“Perhaps we should have hired more nurses and had some on standby/retainer. When there was a spike [in cases], the procurement, HR and finance procedures were burdensome to hire more staff quickly enough. When they finished working through that process, the spike was over.” (FGD, IMC South Sudan field study team)*

#### Data quality assurance practices

An immediate challenge for the study was that training and all data quality assurance procedures had to be conducted entirely remotely, given HQ investigators were unable to travel due to COVID-19. Several steps taken during implementation were considered enabling factors that strengthened data quality during the study, despite the travel limitations. JHSPH, CDC, and IMC headquarters staff conducted a thorough training of country-based research staff, delivered remotely over eight days of half-day sessions on study protocols and data collection forms. Country-based teams subsequently staggered this training to data collectors through in-person sessions, accompanied by a hands-on training in the use of equipment. Study staff emphasized the smaller, in-person, hands-on training helped to standardize clinical measurements and reporting.

*“Another good practice was the practical session with MoH staff included during the stepdown training conducted by the DRC team where they practiced anthropometric measurements. These practicing measurements done in the beginning of the study, probably helped the accuracy of the anthropometric data. It also permitted consensus on the measurement approach.” (FGD, IMC South Sudan field study team)*

Field-based research staff in both countries broadly agreed the remote training was effective and contributed to stronger data quality. They also appreciated the refresher training offered halfway through the data collection period, which focused on addressing routinely observed data entry and measurement problems. In DRC, the research team repeated the training with MoH staff at each enrollment site and included practical sessions which reportedly improved data quality.

Daily and weekly reviews of data quality by the in-country team and remote team, further reportedly improved data quality. These reviews considered missing data, implausible data, enrollment, or follow-up on patients not according to the study protocol, and inconsistencies between the excel-based line list and the study’s data management platform. Potential errors were exhaustively reviewed and rectified, including re-measurement of clinical variables where feasible.

#### Coordination arrangements

Study staff highlighted the importance of coordination and collaboration with local stakeholders. The DRC team believed regular communication, early engagement of stakeholders, and Memorandums of Understanding (MoUs) helped to facilitate the participation of health facilities as enrollment sites. IMC in DRC and SSD coordinated early with the relevent Ministries to secure their support with ethical reviews, protocol development, and identification of study sites. In addition, IMC disseminated study briefing sheets and presented interim findings to the MoH and other stakeholders with the aim of informing health authorities about the ongoing study. In DRC, MoH staff were active partners in data collection during the study, with three government-run inpatient facilities serving as enrollment sites. In SSD, MoH buy-in for the study helped to secure the collaboration of the rapid response team providing home-based case management. Finally, the Ministries in both countries encouraged laboratories to share patient test results with IMC, which reduced delays in recruitment of eligible cases and increased the pace of enrollments per protocol.

## Discussion

This case study was developed to reflect on the complex operational environment in which a COVID-19 prospective cohort study was conducted within two humanitarian settings in Sub-Saharan Africa. The operational challenges and good practices identified–primarily in relation to participant recruitment and study management–were in general not specific to COVID-19, and have been identified in previous research carried out in the midst of epidemics [5–8]. However, the global impact of COVID-19, its high public profile, and specific pandemic policies appear to have exacerbated these problems and contributed to a final sample that underrepresented lower income individuals and nationals, and resulted in lower rates of enrollment than planned.

Testing capacity in both countries impacted the cohort study’s final sample in terms of the number and types of cases enrolled. The worldwide demand for COVID-19 tests strained supply chains and resulted in shortages in testing supplies, particularly in South Sudan. This had a particular impact on enrollments among those who could not afford fees to test in private labs and resulted in high levels of testing among travelers relative to the general population. Disproportionate testing among travelers mirrored trends in enrollment we observed in the cohort study (fully described in companion papers), where 43.0% (n = 211) of people enrolled cited travel as the reason for testing and 23.2% (n = 121) of enrollments were among non-nationals of the country in which the testing was conducted (S1 Table). Travelers were less likely to be symptomatic (52.6% v. 88.9%, p<0.001) and less likely to be hospitalized (19.0% v. 35.0%, p<0.001) [9]. This could have affected representativeness of our study cohort through oversampling of less severe cases.

Our study’s enrollment figures also mirror KI perceptions of demographic disparities in testing and case identification. The proportion of younger people and females enrolled in our study is notably lower than each country’s population distribution (S2 Table). Mandatory testing for travel has in general not been a feature of infectious disease research prior to COVID-19 and had unique implications for our study. The travelers enrolled in the study were more likely to be asymptomatic, adult males, and of higher socio-economic status. As a result, these groups were over-represented in our sample compared to the population in need of humanitarian assistance who were the study’s target population.

Stigmatization of COVID-19 individuals also negatively affected our study; KIs identified negative perceptions of the virus as the primary influencer of demand for testing, careseeking, and willingness to engage with the public health system including researchers. Stigma against infected individuals has similarly influenced research on Ebola, HIV, SARS, Zika, and other infectious diseases [6, 16, 17]. The intense global attention on COVID-19 may have had an adverse effect on the general public’s willingness to test and seek care, which is consistent with the findings of reduced COVID-19 careseeking behaviors reported in other research [18–20].

A high proportion of cases referred to our study either refused to enroll (6.5%) or were unreachable (51.1%), which is consistent with the challenges in recruitment and retention of participants reported from humanitarian research elsewhere [6]. Among the 2,446 eligible cases reported in the study area, more than half (51.1%) were unreachable after three attempts. Unreachable cases often had provided incomplete or incorrect contact information. The situation was particularly difficult in SSD where a large majority (76.7%) of cases were unreachable and the rate of refusals was 8.4%. Ultimately only 21.6% of eligible cases were enrolled, with a much higher enrollment rate in DRC than SSD (31.7% vs. 14.4%) (S3 Table). Enrollment statistics are more fully described in the companion papers [9, 10].

Remote study management has been a routine feature of pandemic research, and we proactively planned for remote management and its associated challenges from the design stage. While the primary investigators were unable to travel to the study locations, research managers were recruited in each country to oversee data collection and stakeholder coordination. We found this approach particularly effective in SSD, where the study utilized only one inpatient facility compared to three inpatient facilities in DRC that were geographically dispersed across insecure areas. Data quality challenges were mitigated through several remote trainings and strict data quality oversight practices. A previous IMC study in SSD [21] identified remote monitoring of data quality as an important factor in the study’s success during a period of active conflict, a practice that we repeated with large success. However, we experienced more challenges in ensuring data quality in DRC compared to SSD. This was partly due to lack of French-language skills among the headquarters-based researchers, which slowed communication. Moreover, the inpatient facility in SSD was directly run by IMC staff, while in DRC the study sites were MoH facilities and much of the data was collected through MoH staff. More complex data collection and reporting arrangements in DRC exacerbated the data quality challenges relative to SSD.

We also encountered various data quality challenges related to clinical measurements. In particular, the study’s research nurses were regularly unable to take anthropometric measurements on critically ill patients because of sensitivities and challenges in measuring these variables. Missing data particularly affected nutritional status indicators, with 6.9% of patients missing data on weight, 6.6% on height, 6.0% MUAC, and 59.0% hemoglobin concentration–with relateively large differences in missingness among hospitalized versus non-hospitalized patients (S4 Table). Another challenge related to measurement was that the study relied primarily on routine clinical protocols and patient-self report for the assessment of many comorbidities, including malaria, HIV, and TB. However, limited laboratory testing was carried out among COVID-19 patients, and other immunocompromising infections were rarely self-reported. The final sample size of patients with comorbidities was small and there was insufficient power to fully explore associations with these variables [9, 10].

Other research has highlighted the need for adaptability and contingency planning in studies undertaken in epidemics [6]. KIs described several positive examples of proactive decision-making during our study. For example, when low case numbers were observed in one of the original DRC study sites in the first month of data collection, the team quickly established new partnerships with facilities that admitted a larger number of COVID-19 patients. Given the innate difficulties with predicting the geography, volume and evolution of COVID-19 cases, these adjustments likely boosted the final sample size.

**Fig 2:**
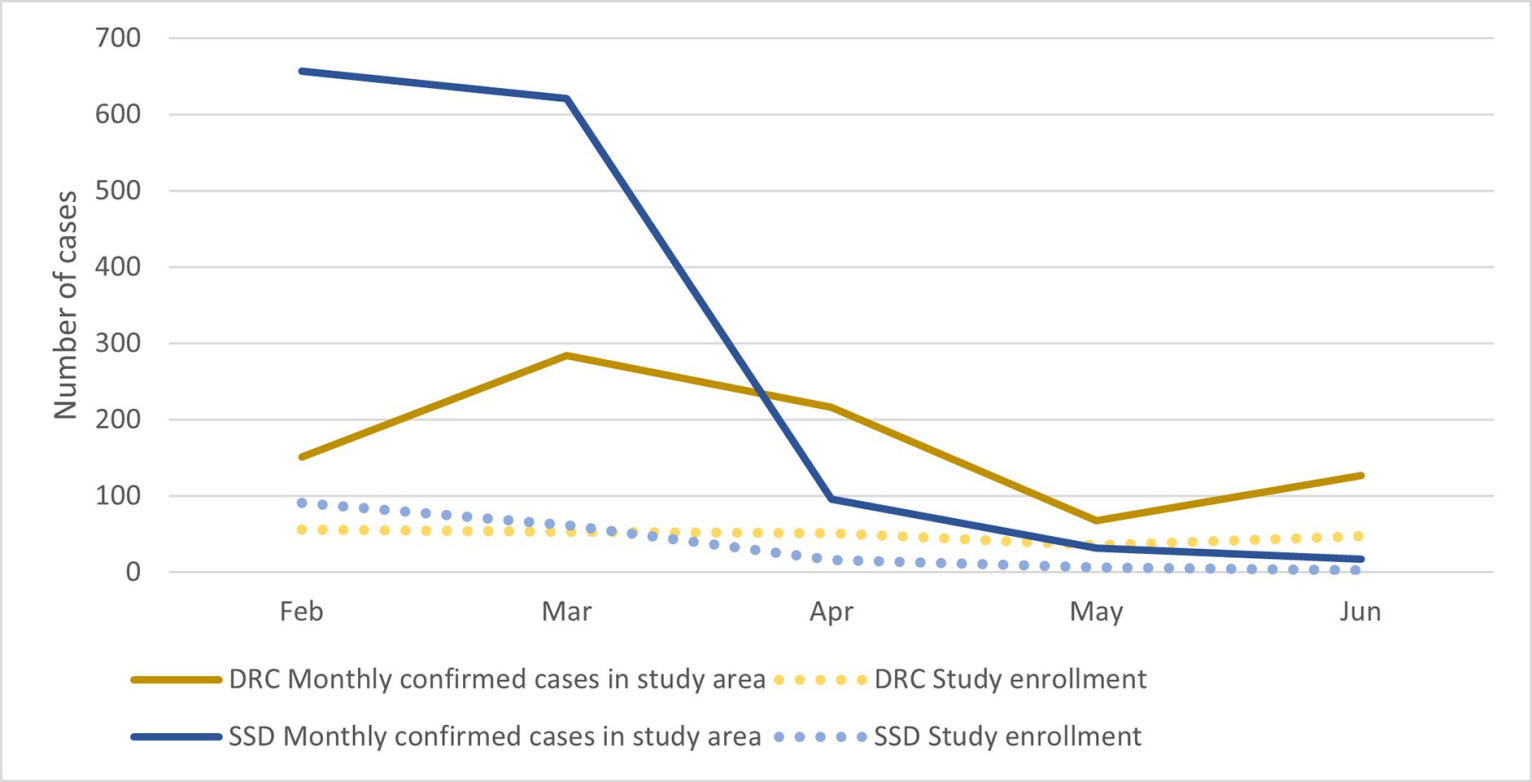
Monthly trends in new cases reported in study area compared to participant enrollment* in SSD, December 2020-June 2021. SSD = South Sudan *Systematic tracking of eligible cases in SSD through laboratory results began Feb. *2021*. *Data prior to this period is not reported in this figure*.

On the other hand, we were less successful in adapting human resource capacity to increase enrollments during periods of higher infections. Trends in enrollment into the study generally mirrored the trends in caseload in each country (Fig 2). However while the highest level of enrollment in SSD occurred in February 2021 during the country’s second wave, the study team struggled to take full advantage of the rise in cases and only enrolled a small proportion of the total cases reported during the wave.

The secondment of staff from other departments improved enrollment rates somewhat; however, enrollments did not increase enough to achieve the target sample size (n = 1,000), which should have been possible given that each country experienced waves with increased cases in the study areas during the data collection period. The staffing structure in each country remained static throughout the study period, and organizational procedures limited our ability to rapidly mobilize additional data collectors to absorb the rise in cases.

Finally, partnerships with local organizations and health structures have been pivotal to successful research in complex emergency environments [17, 21–24]. Engagement with Ministries of Health in SSD [21] and DRC [24] has been a feature of previous IMC research in these settings. Our study similarly benefited from strong collaboration with Ministries of Health, which facilitated data sharing arrangements with laboratories, study site participation, and overall acceptance of the study. We anticipate these coordination mechanisms will support uptake of findings from the study to inform policy and clinical practice where relevant.

## Limitations

This case study was subject to a few limitations. First, not all themes and sub-themes identified could be included in the analysis, resulting in some key challenges being potentially missed or not discussed. Second, although the KIIs and persons included in the FDGs were chosen strategically, it is possible that a person with important insights such as someone with more experience with outpatient care was not included. Third, the challenges discussed are based on study settings in SSD and eastern DRC and may not be generalizable to other humanitarian emergency research settings. Lastly, some challenges were unique to the individual study sites so may not have been included in the analysis as broader, comprehensive themes were the focus.

## Conclusion

Tremendous efforts have gone into describing the impacts of COVID-19 in Africa and addressing research gaps in African settings throughout the pandemic. The operational complexities confronting COVID-19 research have thus far been less well documented, particularly in humanitarian settings. We experienced many similar challenges to those reported in previous research on infectious diseases in humanitarian settings, including under-resourced health systems, limited diagnostic capacity, and limited demand for testing and careseeking due to social stigmatization. However, the nature of the pandemic intensified problems with diagnostic testing and participant recruitment, resulting in a smaller than anticipated sample of patients that was also heavily biased towards travelers.

We identified several important enabling factors for COVID-19 research and other infectious disease research in humanitarian settings through this case study. These considerations, while not exhaustive, may strengthen the quality of future studies. Predicting case trends in a rapidly evolving pandemic is challenging, and the quality of studies will benefit from donors allowing researchers flexibility to extend the timeline for data collection, increase funding to expand study sites, or increase the number of data collectors during waves of increased infections in order to achieve the required sample size. Flexibility in budget lines would further aid this. Staffing plans for research in epidemics / pandemics should include flexibility to quickly scale up or down the study team. Flexible contracting mechanisms such as part time casual positions or leveraging established relationships with local universities that would enable students to act as data collectors could be successful strategies to improve surge capacity in future studies.

Researchers should anticipate low levels of testing through national systems, as well as differential access to and demand for testing among the target population of people in need of humanitarian assistance. As much as possible, study design should include strategies to improve coverage of testing, for example developing agreements with laboratories to pay fees for study participant testing, or to systematically test suspected cases (e.g., with rapid tests for suspected morbidities such as malaria and COVID-19) among the study population who are outpatients. Finally, it is worth reiterating the importance of early collaboration with in-country stakeholders that other researchers have identified. Future studies will benefit from establishing similar partnerships especially with health authorities, prospective study sites, laboratories, and other health program implementers. These local partnerships had a positive influence on our study in terms of its acceptability, operational arrangements, and opportunities for results uptake.

## Supporting information

S1 Table Characteristics of patients in cohort study, by country and location of enrollment. DRC = Democratic Republic of Congo, SSD = South Sudan.
(DOCX)

S2 Table Demographic characteristics of patients in cohort study, by country and location of enrolment. DRC = Democratic Republic of Congo, SSD = South Sudan.
(DOCX)

S3 Table Enrollment results among confirmed patients approached for participation in cohort study. DRC = Democratic Republic of Congo, SSD = South Sudan.
(DOCX)

S4 Table Missing nutritional data among patients enrolled in cohort study. MUAC = Mid-Upper Arm Circumference, HBA1C = Hemoglobin A1c.
(DOCX)
