# Supplementary material for: Operational challenges and considerations for COVID-19 research in humanitarian settings: A qualitative study of a project in Eastern Democratic Republic of the Congo and South Sudan
Source: PLoS One. 2022 Jun 30;17(6):e0267822. doi: 10.1371/journal.pone.0267822 (PMC9246222; doi:10.1371/journal.pone.0267822)
Supplement: S1 Table — DRC = Democratic Republic of Congo, SSD = South Sudan. (DOCX) [file pone.0267822.s001.docx]

**S1 Table. Characteristics of patients in cohort study, by country and location of enrollment^a^**

|  | **All participants** | **By country** | | |
| --- | --- | --- | --- | --- |
|  |  | DRC | S Sudan | p-value |
|  | N (%) | N (%) | N (%) |  |
| **Nationality** |  |  |  | <0.001 |
| National | 395 (76.6%) | 127 (88.2%) | 268 (72.0%) |  |
| Other African country | 68 (13.2%) | 7 (4.9%) | 61 (16.4%) |  |
| Non-African Country | 53 (10.3%) | 10 (6.9%) | 43 (11.6) |  |
| **Reason for COVID-19 test** |  |  |  | <0.001 |
| COVID-19 symptoms | 196 (39.9%) | 84 (60.9%) | 112 (31.7%) |  |
| Known exposure | 71 (14.5%) | 12 (8.7%) | 59 (16.7) |  |
| Travel | 211 (43.0%) | 40 (29.0%) | 171 (48.4%) |  |
| Other | 13 (2.6%) | 2 (1.4%) | 11 (3.1%) |  |

^a^ Produced using data from cohort study described in companion papers [9-10]
