# Supplementary material for: Operational challenges and considerations for COVID-19 research in humanitarian settings: A qualitative study of a project in Eastern Democratic Republic of the Congo and South Sudan
Source: PLoS One. 2022 Jun 30;17(6):e0267822. doi: 10.1371/journal.pone.0267822 (PMC9246222; doi:10.1371/journal.pone.0267822)
Supplement: S2 Table — DRC = Democratic Republic of Congo, SSD = South Sudan. (DOCX) [file pone.0267822.s002.docx]

**S2 Table. Demographic characteristics of patients in cohort study, by country and location of enrollment^a^**

|  | **All participants** | **By Enrollment Country** | | | **National Demographic Characteristics^b^** | |
| --- | --- | --- | --- | --- | --- | --- |
|  |  | DRC | SSD | p-value |  |  |
|  | N (%) | N (%) | N (%) |  | DRC | SSD |
| **Age Category** (years) | 21 (4.0%) | 20 (7.6%) | 1 (0.4%) | <0.001 | 52.7% | 48.5% |
| <18 |  |  |  |  |  |  |
| 18-44 | 308 (59.3%) | 139 (53.1%) | 169 (65.8%) |  | 35.8% | 38.3% |
| 45-64 | 147 (28.3%) | 77 (29.4%) | 70 (27.2%) |  | 9.0% | 10.6% |
| >65+ | 43 (8.3%) | 26 (9.9%) | 17 (6.6%) |  | 2.5% | 2.6% |
| **Sex** | 346 (66.7%) | 153 (58.4%) | 193 (75.1%) | <0.001 | 50.0% | 51.4% |
| Male |  |  |  |  |  |  |
| Female | 173 (33.3%) | 109 (41.6%) | 64 (24.9%) |  | 50.0% | 48.6% |

^a^ Produced using data from cohort study described in companion papers [9-10]

^b^ Source: US Census Bureau International Database, 2021 population estimates
