# Supplementary material for: Operational challenges and considerations for COVID-19 research in humanitarian settings: A qualitative study of a project in Eastern Democratic Republic of the Congo and South Sudan
Source: PLoS One. 2022 Jun 30;17(6):e0267822. doi: 10.1371/journal.pone.0267822 (PMC9246222; doi:10.1371/journal.pone.0267822)
Supplement: S3 Table — DRC = Democratic Republic of Congo, SSD = South Sudan. (DOCX) [file pone.0267822.s003.docx]

**S3 Table. Enrollment results among confirmed patients approached for participation in cohort study^a^**

|  | **Eligible Individuals** | **Enrollment Country** | | |
| --- | --- | --- | --- | --- |
|  |  | DRC | SSD |  |
|  | 2,446 (100%) | 1,023 (100%) | 1,423 (100%) |  |
| **Enrollment status**  Accepted / Enrolled | 529 (21.6%) | 324 (31.7%) | 205 (14.4%) |  |
| Refused | 158 (6.5%) | 38 (3.7%) | 120 (8.4%) |  |
| Unreachable | 1,251 (51.1%) | 160 (15.6%) | 1,091 (76.7%) |  |
| Residence outside of study area | 264 (10.8%) | 264 (25.8%) | 0 (0.0%) |  |
| Died before enrollment attempt | 24 (1.0%) | 24 (2.3%) | 0 (0.0%) |  |
| Results received late | 213 (8.7%) | 213 (20.8%) | 0 (0.0%) |  |
| Disputed test result | 7 (1.3%) | 0 (0.0%) | 7 (0.5%) |  |

^a^ Produced using data from cohort study described in companion papers [9, 10]
