# Supplementary material for: Operational challenges and considerations for COVID-19 research in humanitarian settings: A qualitative study of a project in Eastern Democratic Republic of the Congo and South Sudan
Source: PLoS One. 2022 Jun 30;17(6):e0267822. doi: 10.1371/journal.pone.0267822 (PMC9246222; doi:10.1371/journal.pone.0267822)
Supplement: S4 Table — MUAC = Mid-Upper Arm Circumference, HBA1C = Hemoglobin A1c. (DOCX) [file pone.0267822.s004.docx]

**S4 Table. Missing nutritional data among patients enrolled in cohort study^a^**

|  | All patients | **Missing values, by Status** | | |
| --- | --- | --- | --- | --- |
|  |  | Ever Hospitalized | Non-Hospitalized | p-value |
|  | N (%) | N (%) | N (%) |  |
| **Nutritional status variables** |  |  |  |  |
| Weight | 36 (6.9%) | 31 (21.2%) | 5 (1.3%) | <0.001 |
| Height | 34 (6.6%) | 31 (21.2%) | 3 (0.8%) | <0.001 |
| MUAC | 31 (6.0%) | 23 (15.8%) | 8 (2.1%) | <0.001 |
| HBA1C^b^ | 12 (30.0%) | 11 (39.3%) | 1 (8.3%) | 0.067 |
| Hemoglobin | 306 (59.0%) | 86 (58.9%) | 220 (59.0%) | 0.990 |

^a^ Produced using data from cohort study described in companion papers [9, 10]

^b^ Among cases reporting a history of diabetes only
